# Molecular determinants that govern scaRNA processing by Drosha/DGCR8

**DOI:** 10.1242/bio.054619

**Published:** 2020-10-26

**Authors:** Douglas M. McLaurin, Madelyn K. Logan, Katheryn E. Lett, Michael D. Hebert

**Affiliations:** Department of Cell and Molecular Biology, The University of Mississippi Medical Center, Jackson, MS 39216-4505, USA

**Keywords:** Cajal body (CB), Small Cajal body-specific RNA (scaRNA), MicroRNA (miRNA)

## Abstract

The Cajal body (CB) is a subnuclear domain that participates in the biogenesis of many different types of ribonucleoproteins (RNPs), including small nuclear RNPs (snRNPs), small Cajal body-specific RNPs (scaRNPs) and telomerase. Most scaRNAs, the RNA component of scaRNPs, accumulate in CBs. However, there are three scaRNAs (scaRNA 2, 9, and 17) that are known to be processed into small, nucleolar-enriched fragments. Evidence suggests that these fragments are packaged into a new class of RNPs, called regulatory RNPs (regRNPs), and may modify small nucleolar RNP (snoRNP) activity, thus playing a role in rRNA modification. However, the mechanism by which these fragments are produced is unknown. Previous work has reported the involvement of Drosha and DGCR8 in the cleavage of primary-scaRNA9. Here, we expand on that knowledge by identifying sequence elements necessary for the efficient production of these RNA fragments and demonstrate that primary scaRNA 2 and 17 are also processed by the Drosha-DGCR8 complex. Collectively, our work establishes new factors in the scaRNP biogenesis pathway and adds to the ever-expanding list of noncanonical functions for the microprocessor complex.

## Abstract (precis)

**Summary:** Our findings clearly establish that the Drosha/DGCR8 complex processes a subset of scaRNAs, and further strengthens the connection between CBs and the miRNA processing machinery.

## INTRODUCTION

The Cajal body (CB) is a subnuclear domain that participates in the biogenesis of many different types of ribonucleoproteins (RNPs), including small nuclear RNPs (snRNPs), small Cajal body-specific RNPs (scaRNPs) and telomerase (Meier, 2017). CBs are easily detected in neuronal cells and transformed cells, but are less abundant in other cell types (Sawyer et al., 2016). In cell types lacking CBs, the activities that take place in the CB occur in the nucleoplasm, but at a reduced efficiency compared to cells with CBs (Nizami et al., 2010; Sawyer et al., 2016). One of the activities that take place in the CB is the modification of the small nuclear RNA (snRNA) component of snRNPs. Like rRNA, snRNAs contain numerous modifications such as ribose methylation and pseudouridylation, and these modifications are crucial for ribosomal and snRNP function (Bohnsack and Sloan, 2018; Sloan et al., 2017). In rRNA, ribose methylation and pseudouridylation modifications are conducted by small nucleolar RNPs (snoRNPs) (Sloan et al., 2017). There are two classes of snoRNPs, which are defined by conserved *cis* elements present in the snoRNA. Namely, box C/D snoRNAs form box C/D snoRNPs and box H/ACA snoRNAs form box H/ACA snoRNPs. Box C/D snoRNPs contain fibrillarin (which conducts ribose methylation) and box H/ACA snoRNPs contain dyskerin (which conducts pseudouridylation) (Burke et al., 2018; Lykke-Andersen et al., 2018). These modifications are guided by base pairing of the snoRNA to rRNA. Similarly, the snRNA component of snRNPs is modified by scaRNPs. There are three different classes of scaRNPs defined by conserved *cis* elements present in the scaRNA: box C/D contains fibrillarin (which conducts ribose methylation), box H/ACA contains dyskerin (which conducts pseudouridylation), and mixed domain, which contains both fibrillarin and dyskerin (conducting ribose methylation and pseudouridylation) (Burke et al., 2018). Modifications are guided by scaRNA base pairing to snRNA. An additional *cis* element present in box H/ACA scaRNAs is the body localization (CAB) motif. The CAB motif is bound by the protein WRAP53 (TCAB1/WDR79) to target this class of scaRNA, which includes the telomerase RNA component, to the CB (Izumikawa et al., 2019; Tycowski et al., 2009). It is unclear how box C/D scaRNAs, which lack a CAB motif, accumulate in the CB but TDP-43 may be involved (Izumikawa et al., 2019).

Most scaRNAs and snoRNAs are encoded in the introns of host genes. However, two scaRNAs (scaRNA2 and scaRNA17) are independently transcribed (Gerard et al., 2010; Tycowski et al., 2004). Very interestingly, scaRNA 2 and 17 can be further processed to generate fragments 70–80 nt in length (Tycowski et al., 2004). In addition to scaRNA 2 and 17, scaRNA9 has also been shown to undergo processing, generating two fragments of similar size to that observed for scaRNA 2 and 17 (Tycowski et al., 2004). These fragments mostly accumulate in the nucleolus, but are also localized in the nucleoplasm (CB) (Tycowski et al., 2004). The function of these stable fragments is unclear. It is possible that the processing of primary-scaRNA 2, 9 and 17 is a method to regulate snRNA modifications and hence govern snRNP biogenesis. For example, full-length scaRNA 2, 9 and 17 localize to the CB and there guide modifications present in U2, U4 and U12 snRNAs. The processing of primary-scaRNA 2, 9 and 17 into fragments alters the amount of full-length scaRNA 2, 9 and 17 present in the CB, which in turn would be expected to impact snRNA modification. Alternatively, it is also possible that the fraction of scaRNA 2, 9 and 17-derived fragments that accumulate in the nucleolus interact with snoRNPs and regulate their activity. In other words, some of the fragments generated from scaRNA 2, 9 and 17 may form regulatory RNPs (regRNPs), and we have published data in support of this possibility (Burke et al., 2018; Burke et al., 2019; Poole et al., 2017). Efforts to understand the mechanisms that generate scaRNA 2, 9 and 17 fragments are therefore important to both snRNP and ribosome biogenesis.

We have recently published a paper demonstrating that Drosha and DGCR8, well-characterized components of the miRNA processing pathway (Han et al., 2006; Macias et al., 2013; Nguyen et al., 2015), take part in the biogenesis of fragments derived from primary-scaRNA9 (Logan et al., 2020). In addition, we have shown that CBs can associate with a miRNA gene cluster (the chromosome 19 microRNA cluster, C19MC) and positively regulate miRNA biogenesis (Logan et al., 2020). These findings demonstrate a functional relationship between CBs and the miRNA processing machinery. Here we report that Drosha and DGCR8 also take part in the biogenesis of fragments derived from primary-scaRNA2 and primary-scaRNA17. We also define sequence elements in primary-scaRNA 2, 9 and 17 that are important molecular determinants that regulate processing by Drosha and DGCR8. These findings clearly establish that some scaRNAs are substrates for the Drosha–DGCR8 complex, and further strengthen the connection between CBs and the miRNA processing machinery.

## RESULTS

### Processed scaRNAs

**Fig. 1.**
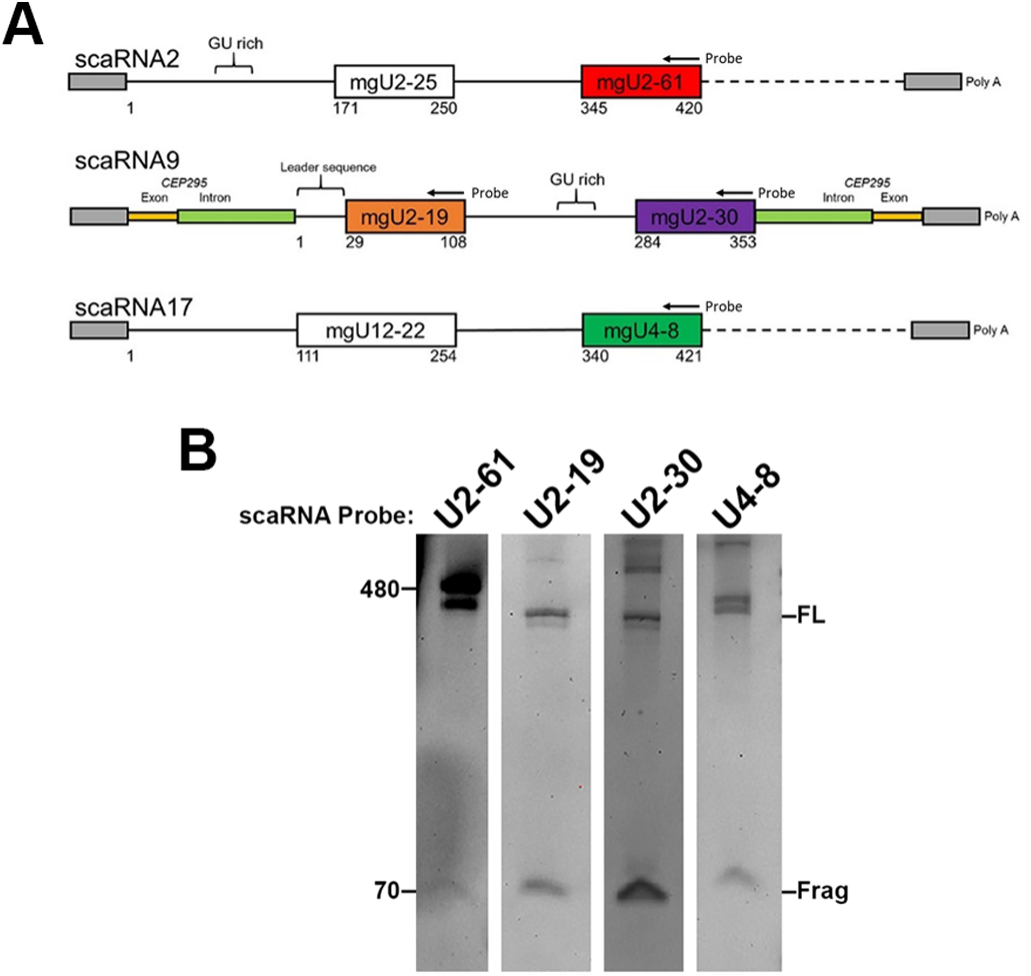
**ScaRNA 2, 9 and 17 expression constructs and processing.** (A) Schematic of expression constructs used to evaluate *in vivo* processing of scaRNA 2, 9 and 17. Sequences encoding these scaRNAs were cloned into pcDNA3.1+. Transcription will generate RNAs that include vector sequences at the 5 ′ and 3 ′ ends of the RNA (gray rectangles), including the addition of a poly A tail. The solid black line represents the endogenous full-length (FL) scaRNA detectable by Northern blotting. The dashed line represents sequences downstream of the FL scaRNA that are important for processing. The colored rectangles denote processed endogenous fragments for each scaRNA that are detectable by Northern blotting. In contrast to scaRNA 2 and 17, which are independently transcribed in the genome, scaRNA9 is encoded in the intron of the *CEP295* host gene. Our pcDNA3.1+scaRNA9 expression construct contains the entire *CEP295* intron that harbors scaRNA9 (green rectangles), along with partial exonic sequences upstream and downstream of this intron (gold rectangles), ensuring proper splicing of the transcribed RNA. The probes used for Northern blotting are indicated. Other notable sequence elements, such as the leader sequence in scaRNA9 and the GU rich regions in scaRNA2 and scaRNA9 (Enwerem et al., 2015; Poole et al., 2016, 2017), are denoted. (B) Detection of ectopically expressed FL and processed fragments by Northern blotting. HeLa cells were transfected with the DNA expression constructions described above, followed by RNA isolation, TBE-Urea gel electrophoresis, Northern blotting and hybridization with probes that bind the indicated region of the scaRNA. The detected signals corresponding to ectopically expressed FL or fragment (frag) for each scaRNA are noted.

Currently, there are three scaRNAs (scaRNA 2, 9 and 17) known to be processed that generate nucleolar-enriched fragments (Tycowski et al., 2004). A schematic of these scaRNAs and fragments is shown in Fig. 1A. White rectangles represent domains that are not processed into smaller fragments. Note that scaRNA 2, 9 and 17 are box C/D scaRNAs, so they guide ribose methylation modifications. Colored rectangles denote the stable stem/loop fragments derived from these primary scaRNAs, and the nomenclature indicates the site of modification in a given snRNA. For example, the mgU2-61 region of scaRNA2 (red rectangle in Fig. 1A) serves as a methylation guide for U2 snRNA at position 61 (Tycowski et al., 2004). Importantly, the stem/loop scaRNA 2, 9 and 17 fragments (Fig. 1A, colored rectangles) are approximately the same size (70–80 nt) (Tycowski et al., 2004) as precursor miRNAs generated by Drosha/DGCR8, indicating that some primary scaRNAs may be unorthodox substrates for the miRNA processing machinery. Our recent work supports this hypothesis (Logan et al., 2020). To further investigate the mechanisms that generate scaRNA 2, 9 and 17 fragments, we have generated expression constructs in the pCDNA3.1 vector, which yield sufficient amounts of ectopically expressed full-length (FL) and fragment (frag) that can be easily detected by Northern blotting using specific DIG-labelled probes (Fig. 1B). In contrast, endogenous scaRNA 2, 9 and 17 fragments are more difficult to detect using non-radioactive probes. Although ectopically expressed scaRNA 2, 9 and 17 can yield the appropriate processed fragments (Fig. 1B), it is important to appreciate that these plasmid constructs do not fully recapitulate endogenous expression conditions. In particular, important *cis* elements that promote or facilitate processing may be lacking in these constructs. Altered expression conditions as a result of ectopic scaRNA 2, 9 and 17 transcription from a pCDNA3.1 vector (Fig. 1A) may account for the observed differences in the amount of processed fragment present for each scaRNA. For example, fragments derived from scaRNA9 (mgU2-19 and mgU2-30) are easily detected whereas the amount of fragment derived from scaRNA2 (mgU2-61) is relatively less abundant considering the high amount of FL scaRNA2 signal (Fig. 1B). It is possible that scaRNA9 fragments are more efficiently generated because scaRNA9 is present within an intron and splicing of this ectopically expressed pre-mRNA allows for scaRNA9 to be appropriately targeted to the Drosha/DGCR8 processing machinery. In contrast, scaRNA2 and scaRNA17 expression from pCDNA3.1 creates a hybrid mRNA consisting of various elements, including a poly A tail, that are not present in endogenous full-length scaRNA 2 and 17. We speculate that these scaRNA 2 and 17 hybrid mRNAs are not efficiently targeted to the Drosha/DGCR8 processing machinery. Inefficient Drosha/DGCR8 processing of ectopically expressed scaRNA 2, 9 and 17 may also result in the accumulation of primary scaRNAs or intermediates that are larger in size than the full-length scaRNAs (Fig. 1B).

### Downstream sequences are important for scaRNA 2 and 17 fragment biogenesis

**Fig. 2.**
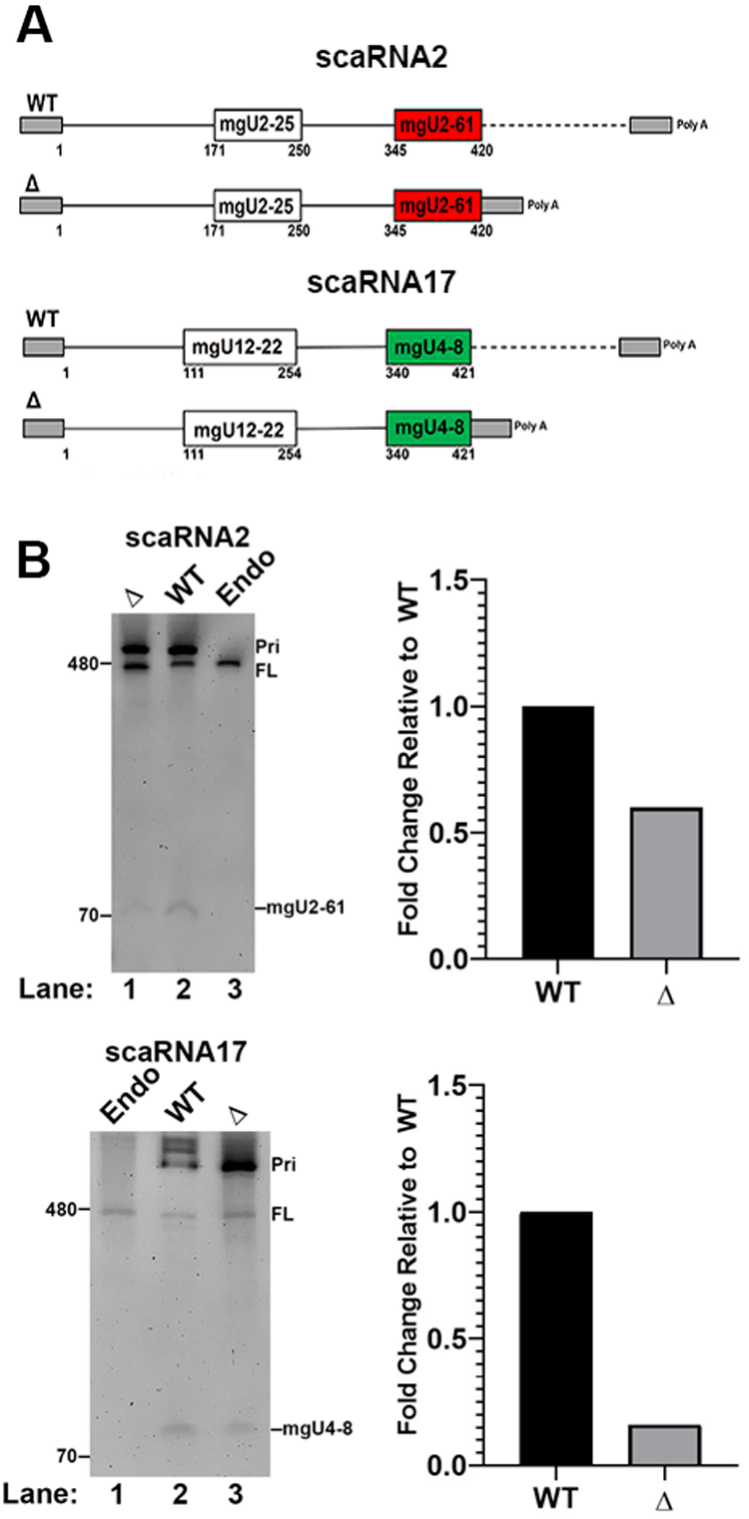
**An extended 3′ sequence is required for the efficient generation of scaRNA fragments.** DNA constructs expressing wild-type (WT) or 3′ deletion (Δ) scaRNA2 or scaRNA17 were transfected into HeLa cells followed by RNA isolation, Northern blotting and detection with probes described in Fig. 1. Full-length (FL) scaRNA 2 and 17 and fragments thereof are indicated. Endogenous signals are present in lanes containing RNA from untransfected cells (Endo). Primary-scaRNA signal is denoted (Pri). The data shown in the gels was quantified and displayed in histograms. Specifically, the fragment signal was divided by the FL+Pri signal and the WT value was set to 1. Both the FL+Pri signals were included in the calculation in order to account for all ectopic expression products.

It is known that, in general, the microprocessor complex identifies the basal junction between single stranded RNA and double stranded RNA in primary-miRNA as a target for processing (Han et al., 2006). Additionally, there are many conserved motifs present in primary-miRNAs that promote processing by Drosha/DGCR8 (Auyeung et al., 2013). To evaluate the importance of single stranded RNA downstream of the processed fragments in scaRNA 2 and 17, we generated expression constructs lacking sequence downstream of the end of the fragments (Fig. 2A). These constructs were transfected into cells and RNA was isolated 24 h later. Northern blotting of isolated RNA showed that deletion constructs had a reduced amount of scaRNA2 and scaRNA17 fragment detected relative to full-length scaRNA compared to that obtained with wild-type (WT) constructs (Fig. 2B, Histograms). These findings demonstrate that sequences downstream of the fragment region of scaRNA 2 and 17 impact fragment processing.

### The scaRNA9 intronic context influences mgU2-30 fragment levels

**Fig. 3.**
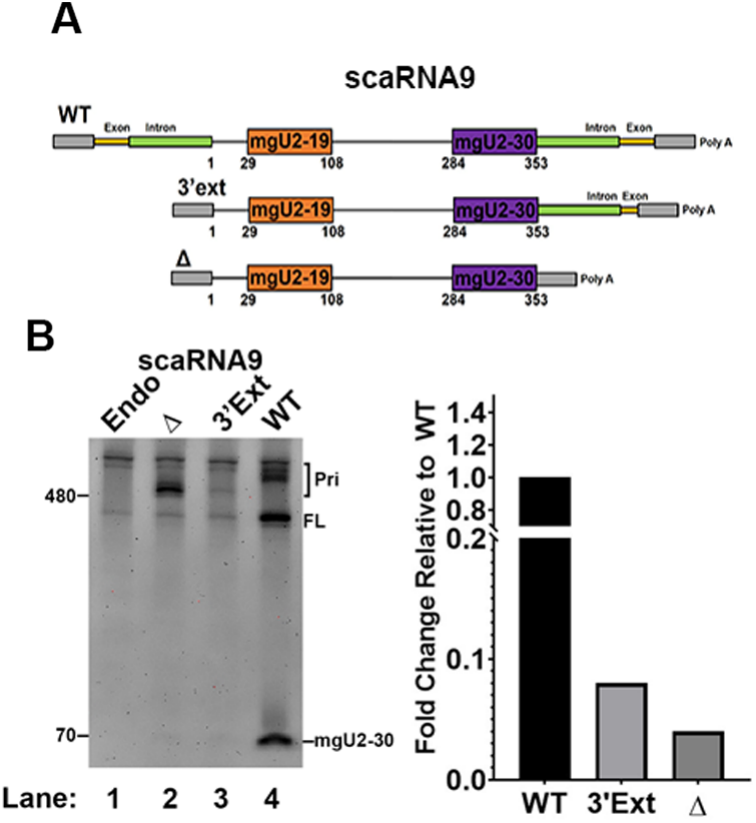
**Intronic sequences are required for the efficient processing of scaRNA9.** (A) Schematic of scaRNA9 constructs illustrating the absence of 5′ intronic sequence in the 3′ extension construct (3′ ext) and the lack of both 5′ and 3′ intronic sequences in the deletion construct (Δ). (B) Detection of full-length (FL) scaRNA9 and the mgU2-30 fragment by Northern blot using RNA from untransfected HeLa cells (Endo) or HeLa cells ectopically-expressing WT, 3′ ext, or Δ scaRNA9. Primary-scaRNA signal is denoted (Pri). The data shown in the gel was quantified (histogram). Specifically, the fragment signal was divided by the FL+Pri signal and the WT value was set to 1. Both the FL+Pri signals were included in the calculation in order to account for all ectopic expression products.

We next investigated the upstream and downstream sequences of scaRNA9 that may impact the biogenesis of the mgU2-30 fragment. Unlike scaRNA2 and scaRNA17, which are independently transcribed, scaRNA9 is encoded in the intron of the *CEP295* host gene (Fig. 1A, Fig. 3A). Gene expression analysis from GTEx (Release V6) which includes 53 tissues (University of California, Santa Cruz, CA, USA, Genome Browser) shows that the *CEP295* host gene is uniformly expressed in most tissues examined. An approximate twofold induction of *CEP295* expression in cerebellar hemisphere, cerebellum and testis was noted relative to most other tissues examined. All of these tissues, therefore, would be expected to produce intronically-encoded scaRNA9. Previous work has shown that miRNA processing of intronically expressed primary-miRNAs can take place before splicing (Janas et al., 2011; Kim and Kim, 2007). Indeed, introns in which the pre-miRNA has been removed by Drosha/DGCR8 are still subjected to splicing (Kim and Kim, 2007). In other words, the intron-encoded primary-miRNA does not need to be excised by the spliceosome before miRNA processing. To determine if the intronic context of scaRNA9 is important for the biogenesis of the mgU2-30 fragment, a DNA expression construct was engineered that deletes the intronic sequence upstream of the scaRNA9 coding sequence, leaving the intronic sequence downstream of the coding region intact (3′ ext) (Fig. 3A). Additionally, we also generated a construct that deletes intronic sequences upstream and downstream of the scaRNA9 coding sequence (Δ) (Fig. 3A). Expression of WT and mutant scaRNA9 constructs in cells, followed by RNA isolation and Northern blotting with a mgU2-30 probe reveals that relatively little mgU2-30 fragment is generated from the upstream and downstream deletion construct (Δ) (Fig. 3B, compare the amount of the mgU2-30 signal in lane 2 to that in lane 4). The amount of mgU2-30 fragment is also reduced compared to WT for the construct lacking sequences upstream of the scaRNA9 coding sequence (3′ ext), although we note that the expression of the primary-scaRNA9-3′ ext transcript is less abundant than the other primary transcripts tested. This finding may indicate that the primary-scaRNA9-3′ ext transcript is less stable than the ectopically expressed WT and (Δ) primary transcripts. The relative lack of mgU2-30 fragment with the Δ construct despite the presence of a sizable amount of the primary-scaRNA9-Δ transcript clearly demonstrates that intronic sequences upstream and downstream of the scaRNA9 coding region are necessary for efficient biogenesis of the mgU2-30 fragment (Fig. 3B, histogram).

### *In vitro* processing of scaRNA2 and scaRNA17 by Drosha/DGCR8

**Fig. 4.**
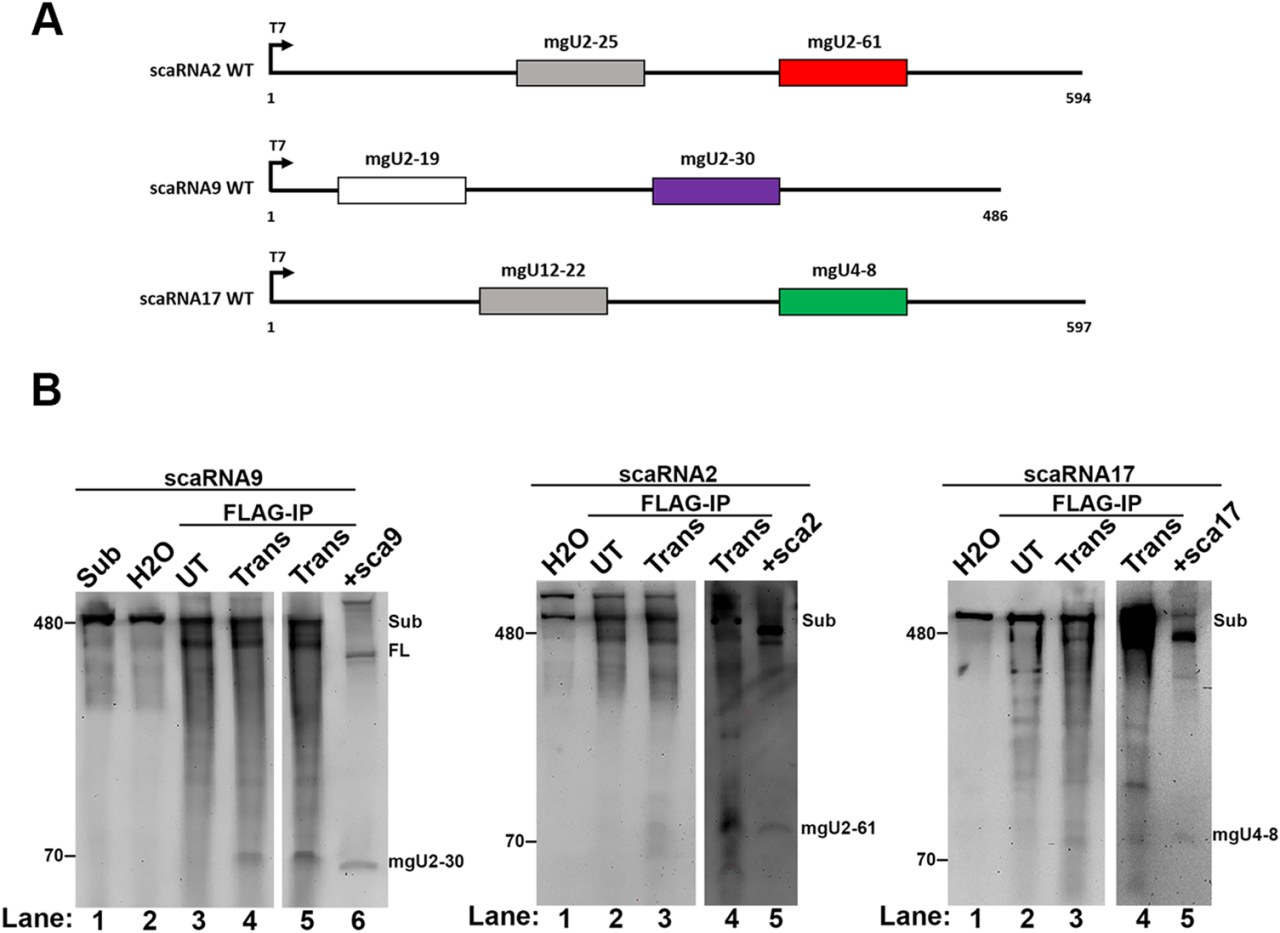
***In vitro* processing of scaRNAs by Drosha/DGCR8.** (A) Schematic of scaRNA 2, 9 and 17 substrates used for *in vitro* processing assays with immunoprecipitated Drosha/DGCR8. Substrates were obtained by *in vitro* transcription of linearized DNA encoding the indicated scaRNA followed by RNA gel purification. (B) *In vitro* transcribed scaRNA9 (left), scaRNA2 (middle) and scaRNA17 (right) substrates were used in a processing assay with immunopurified (FLAG) Drosha/DGCR8. RNA isolated from the processing reactions was subjected to Northern blotting with probes that detect the various full-length scaRNAs and fragments. Substrate was incubated with FLAG beads from Drosha/FLAG-DGCR8 transfected lysate (Trans), FLAG beads from un-transfected cell lysate (UT), or water (H_2_O). Note that two different Trans processing reactions are shown for each substrate (lanes 4 and 5 for scaRNA9 and lanes 3 and 4 for scaRNA2 and scaRNA17). A positive control of ectopically expressed scaRNA9 RNA is shown in the left panel, lane 6 (+sca9). Ectopically expressed scaRNA2 and scaRNA17 are shown in lane 5 of the middle (+sca2) and right (+sca17) panels, respectively.

We have published that Drosha/DGCR8 complexes can process a primary-scaRNA9 substrate *in vitro*, generating both mgU2-19 and mgU2-30 fragments (Logan et al., 2020). We have now examined if Drosha/DGCR8 can generate the mgU2-61 fragment using primary-scaRNA2 and the mgU4-8 fragment using primary-scaRNA17 *in vitro* transcribed substrates in an *in vitro* processing assay (Fig. 4A). As shown in Fig. 4B, processing assays using primary-scaRNA9 substrate incubated with FLAG beads complexed with FLAG-DGCR8/Drosha generate a fragment consistent with mgU2-30 (left panel, lanes 4 and 5), consistent with our previously published results (Logan et al., 2020). A mgU2-30 fragment is not observed when using FLAG beads complexed with lysate from un-transfected cells (left panel, lane 3), or incubated with water alone (lane 2). Full-length scaRNA9 and mgU2-30 fragment from ectopically expressed primary-scaRNA9 is shown in lane 6. Processing assays using FLAG beads from un-transfected cells (UT) and FLAG-DGCR8/Drosha transfected cells (Trans) were also conducted using primary-scaRNA2 (middle panel) and primary-scaRNA17 (right panel). As with primary scaRNA9 (Logan et al., 2020) (Fig. 4B, left panel), fragments corresponding to mgU2-61 (middle panel) and mgU4-8 (right panel) are detected only in reactions using FLAG beads complexed with Drosha/DGCR8 (Trans) and not with FLAG beads from un-transected (UT) cells or incubated with water alone. These *in vitro* generated fragments are approximately the same size as found in cells ectopically expressing scaRNA2 and scaRNA17 constructs (lane 5, middle and right panel, respectively). Hence Drosha/DGCR8 can directly generate fragments from primary-scaRNA 2 and 17 *in vitro*, similar to our previous finding that the microprocessor complex can also generate scaRNA9 fragments (Logan et al., 2020).

### Identification of a mutant in scaRNA9 that disrupts *in vivo* mgU2-30 biogenesis

**Fig. 5.**
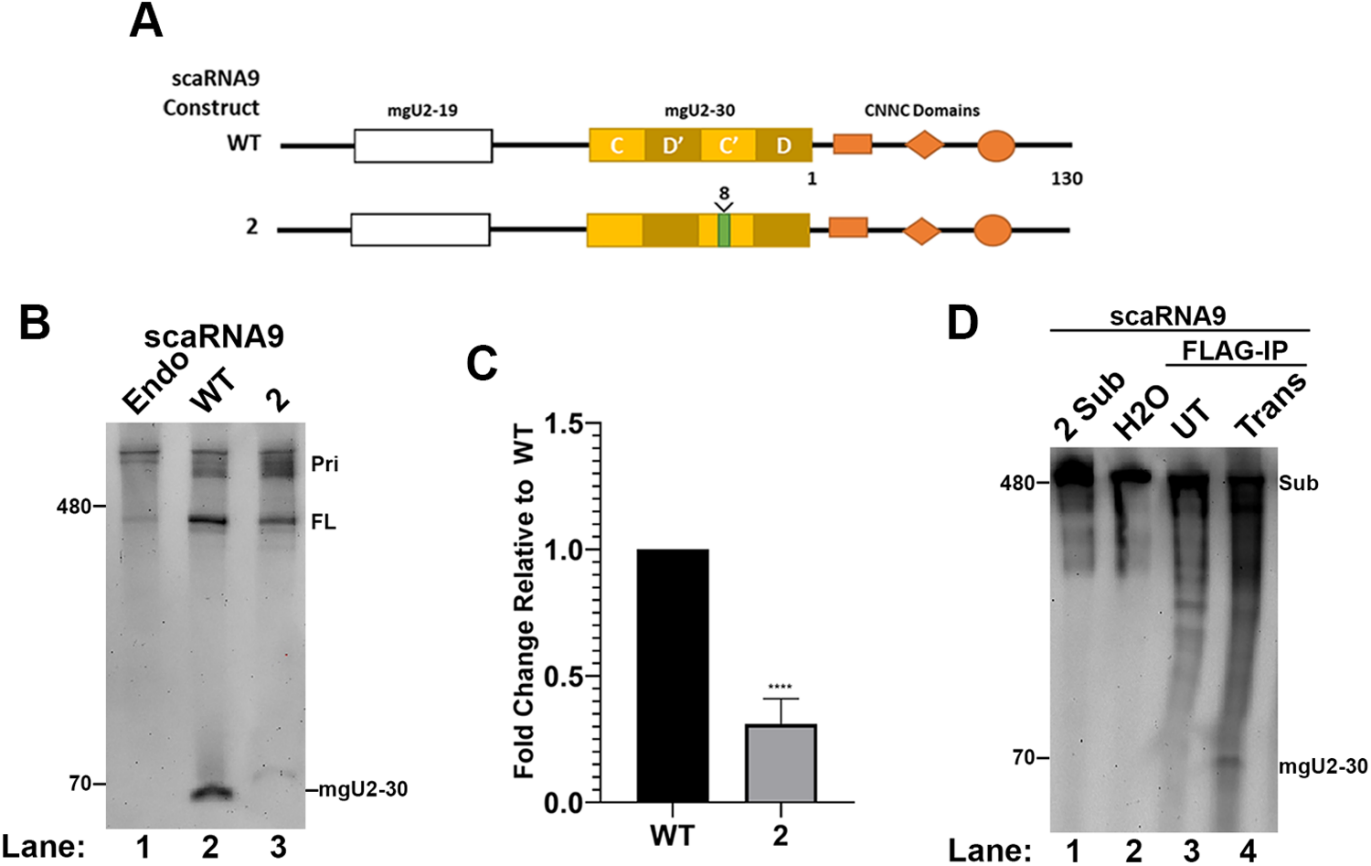
**Disruption of the scaRNA9 C′ box impedes *in vivo* biogenesis of the mgU2-30 fragment.** (A) Schematic of WT and mutant scaRNA9 constructs. The 8 nt insertion in the C′ box of scaRNA9 mutant 2 is shown. Also indicated are CNNC motifs that are known to impact Drosha/DGCR8 processing. (B) Northern blot detection of the mgU2-30 fragment and FL scaRNA9 in RNA isolated from HeLa cells that ectopically express the WT or mutant #2 scaRNA9 constructs. Primary-scaRNA signal is denoted (Pri). (C) Histogram generated from the quantification of data shown in (B). The mgU2-30 signal was divided by the FL scaRNA9 signal in that lane, and normalized to that obtained for WT. *N*=4 biological repeats, ****=*P*<0.0001, error bars represent standard deviation. (D) Northern blot detection of RNA isolated from *in vitro* processing assays with Drosha/DGCR8 and scaRNA9 mutant #2 as the substrate.

Secondary structure predictions of scaRNA9 indicate that the mgU2-19 and mgU2-30 regions form stem/loop structures (Tycowski et al., 2004). The unpaired loop region of these structures is predicted to serve as the guide RNA component in the scaRNP for snRNA modification, in accordance with generally accepted rules as to the function of box H/ACA and box C/D snoRNPs and scaRNPs (Kiss, 2004). To disrupt this stem/loop structure, we have generated a construct with an 8 nt insertion in the C′ motif (Fig. 5A, mutant #2) and tested if this mutation would alter mgU2-30 biogenesis in cells and by *in vitro* processing assays. These constructs were then transfected into cells, followed by RNA isolation, Northern blotting and hybridization with a probe that detects FL scaRNA9 and the mgU2-30 fragment. As shown in Fig. 5B, the amount of the mgU2-30 fragment is reduced relative to FL scaRNA9 when using mutant #2 compared to the fragment/FL ratio observed with the WT scaRNA9 construct. As expected, the mgU2-30 fragment derived from the mutant #2 construct, which contains an 8 nt insertion, migrates slightly slower the WT mgU2-30 fragment. Quantification of this and other data is shown in Fig. 5C. *In vitro* processing assays, however, show that Drosha/DGCR8 complexes are still able to generate the mgU2-30 fragment with the mutant #2 substrate (Fig. 5D).

### Mutation of the downstream region of primary-scaRNA9 decreases *in vivo* mgU2-30 fragment biogenesis

**Fig. 6.**
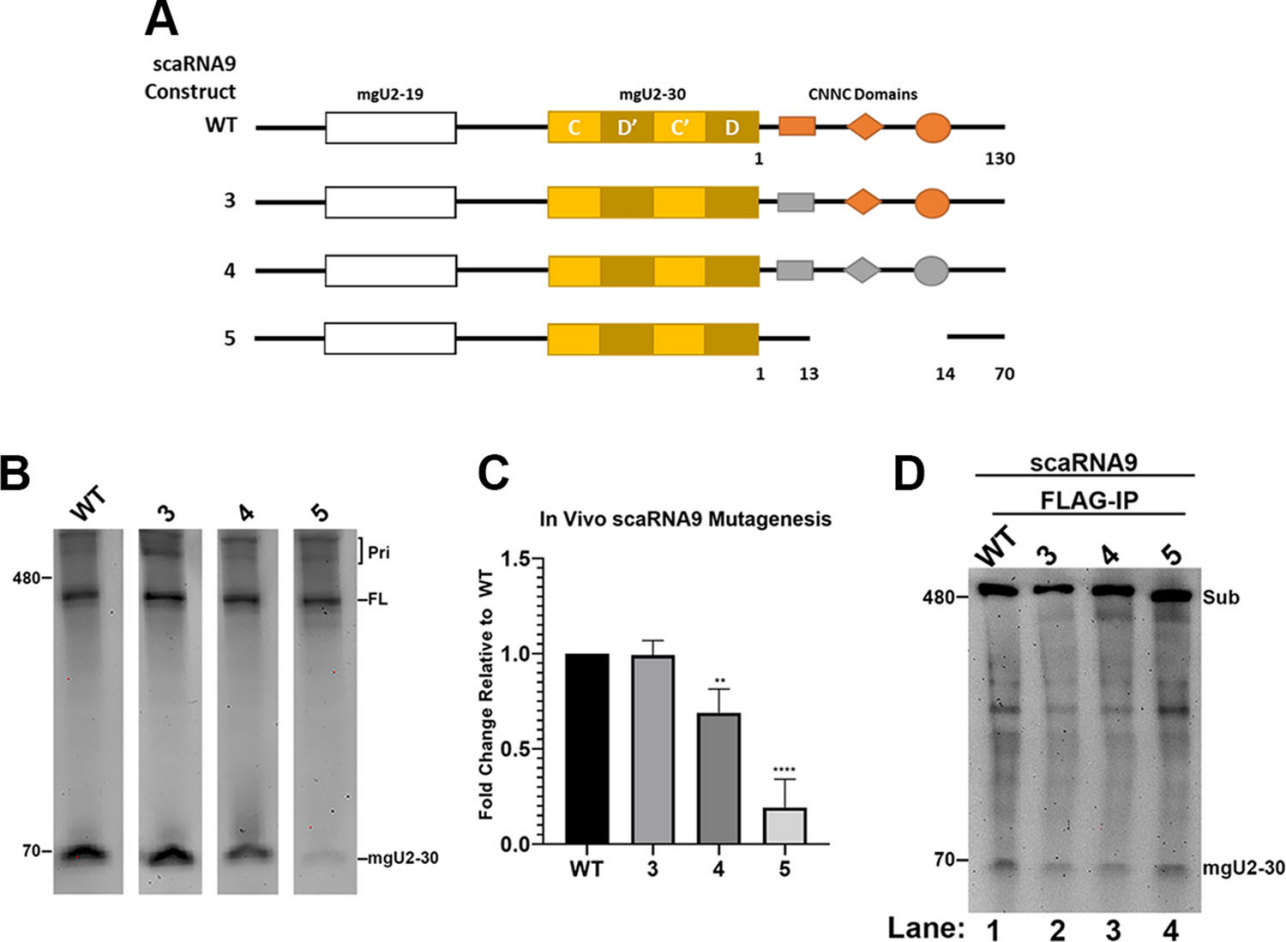
**The CNNC domains impact *in vivo* mgU2-30 biogenesis.** (A) Schematic of scaRNA9 constructs annotating the three CNNC domains found downstream of the mgU2-30 fragment and the mutations that replace or delete these domains in mutants #3, #4, and #5. (B) Northern blot detection of the mgU2-30 fragment and FL scaRNA9 from RNA ectopically-expressing the WT, or mutant #3, #4, or #5 scaRNA9 construct. (C) Histogram generated from the quantification of data shown in (B). For each condition, the mgU2-30 signal was divided by the FL scaRNA9 signal in that lane, and normalized to that obtained from WT. *N*=4 biological repeats, **=*P*<0.0025, ****=*P*<0.0001, error bars represent standard deviation. (D) Northern blot detection of RNA isolated from *in vitro* processing assays with Drosha/DGCR8 using WT and mutant #3, #4 and #5 scaRNA9 constructs as substrates. The mgU2-30 fragment generated by the *in vitro* processing assay is denoted.

It is known that many *cis* elements influence Drosha/DGCR8 primary-miRNA processing efficiency. One of these elements is the CNNC which is located downstream of the basal junction (Creugny et al., 2018). Primary-scaRNA9 has three CNNC motifs in the single stranded region downstream of the mgU2-30 region (Fig. 6A). To monitor if these CNNC motifs impact mgU2-30 biogenesis, we generated three different mutants that progressively disrupt all of the CNNC motifs (mutant #3, mutant #4 and mutant #5 in Fig. 6A). These constructs were then transfected into cells, followed by RNA isolation, Northern blotting, and hybridization with a probe that detects FL scaRNA9 and the mgU2-30 fragment to monitor the *in vivo* processing of these mutated RNAs. As shown in Fig. 6B, and quantified in Fig. 6C, progressive mutation of the scaRNA9 3′ region containing the CNNC motifs decreases the efficiency of mgU2-30 fragment production or stability. Notably, mutant #4, which has the same length of scaRNA9 downstream region as WT scaRNA9 but has mutations in all three CNNC motifs, has a statistically significant reduction in the amount of mgU2-30 fragment relative to FL scaRNA9 compared to that obtained with WT scaRNA9. *In vitro* assays demonstrate that all of the mutants in the scaRNA9 3′ region can still be processed by Drosha/DGCR8 with no dramatic loss of efficiency (Fig. 6D). Collectively, the results of these experiments demonstrate that the CNNC motifs present in the scaRNA9 3′ region impact the *in vivo* biogenesis of mgU2-30. It is likely that the lack of specificity factors in the *in vitro* processing assay account for the observation that scaRNA9 3′ region mutants are still effectively processed by this method, as detailed in the discussion below.

## DISCUSSION

The data presented here expand the number of scaRNAs known to be processed by the Drosha/DGCR8 complex. Our previous work demonstrates that primary-scaRNA9 is a substrate for Drosha/DGCR8 (Logan et al., 2020), and we show here that primary-scaRNA2 and primary-scaRNA17 can also be processed by this complex. Specifically, we show by *in vitro* processing assays that the biogenesis of previously reported scaRNA 2, 9 and 17 fragments (Tycowski et al., 2004) can be achieved by the Drosha/DGCR8 complex. In addition, we identify sequence elements that facilitate scaRNA fragment generation. For example, an important determinant for processing by Drosha/DGC8 is a single stranded RNA region upstream and downstream of the double stranded RNA region (Creugny et al., 2018). This was observed in our previous study, which showed that the mgU2-30 fragment is not efficiently generated in an *in vitro* processing assay when using a scaRNA9 3′ deletion substrate (Logan et al., 2020). Here we report that the 3′ region of both scaRNA2 and scaRNA17, which resides downstream of the processed fragment location, positively influences fragment formation. A previous report (Gerard et al., 2010) also examined the 3′ region of scaRNA2, but this study focused on the role of 3′ region in the formation of full-length scaRNA2 and not the mgU2-61 fragment. Specifically, this report showed that the 3′ region downstream of the end of the scaRNA2 coding region does not impact the formation of full-length scaRNA2 (Gerard et al., 2010). In contrast, we show here that the 3′ region of scaRNA2 does play a role in the processing of primary-scaRNA2 and generation of the mgU2-61 fragment.

We also demonstrate that the 3′ region of scaRNA9 contains CNNC motifs, which are associated with increased processing of primary-miRNAs by Drosha/DGCR8. Mutation of these motifs in primary-scaRNA9 reduces the amount of the mgU2-30 fragment as assayed by *in vivo* processing experiments. Reduced levels of the mgU2-30 fragment were also observed *in vivo* when an 8 nt insertion was placed in the scaRNA9 C′ box, clearly indicating that the Drosha/DGCR8 complex requires an optimal configuration of single stranded RNA regions upstream and downstream of a double stranded RNA region. Interestingly, *in vitro* processing assays using scaRNA9 mutants #2, #4, and #5 do not show a large reduction in the amount of mgU2-30 fragment production as observed in the *in vivo* processing assays. One possible explanation for the difference between our *in vivo* and *in vitro* processing assays is that a specificity factor is present in the *in vivo* assays, which is not part of the Drosha/DGCR8 immuno-complex used in the *in vitro* assays. One such specificity factor could be SRSF3 (SRP20), a splicing factor that has been shown to play a role in the identification of the CNNC motif in primary-miRNAs to help facilitate processing by Drosha (Auyeung et al., 2013; Kim et al., 2018). SRSF3 is not part of the Drosha/DGCR8 immuno-complex, however (Auyeung et al., 2013), so it is likely that DGCR8 can serve in the *in vitro* processing assays as an anchor by binding to the RNA substrate to direct Drosha processing. In contrast, in the environmentally more complex *in vivo* processing assays, SRSF3 likely ensures specificity of Drosha/DGCR8 activity. Collectively, the mutational analysis conducted here expands the known *cis* elements that impact scaRNA 2, 9 and 17 fragment generation, which now includes the leader sequence of scaRNA9, the GU-rich regions in scaRNA 2 and 9, (Enwerem et al., 2015; Poole et al., 2016, 2017), and the 3′ region of scaRNA 2, 9 and 17. Interestingly, the scaRNA9 leader sequence is important for full-length scaRNA9 and mgU2-19 fragment accumulation, but does not impact mgU2-30 fragment levels (Poole et al., 2017). This finding indicates that Drosha/DGCR8 processing of mgU2-30 is not dependent upon mgU2-19 processing.

An important next step will be to assess how the cell regulates the amount of primary-scaRNA 2, 9 and 17 that used to generate full-length scaRNAs that form scaRNPs and localize to the CB versus that which is processed by Drosha/DGCR8 and form RNPs that are primarily nucleolar in localization. Conceivably, the regulation of this cellular decision point will have ramifications for both snRNP biogenesis and rRNA modification. As such, additional studies into the processing of scaRNAs by Drosha/DGCR8 will clarify regulatory mechanisms that impact both the splicing and translation machinery.

## MATERIALS AND METHODS

### Cell lines, plasmids and transfections

HeLa cells were obtained from the American Type Culture Collection (ATCC). Cells were cultured as previously described (Enwerem et al., 2014). ScaRNA2 pcDNA3.1+ (Enwerem et al., 2014), scaRNA9 pcDNA3.1+ (Enwerem et al., 2015), and scaRNA17 pcDNA3.1+ (Poole et al., 2017) have been previously described and are represented by Δ in this manuscript. For WT or 3′ ext constructs, scaRNAs were amplified from genomic DNA isolated from HeLa-ATCC using standard molecular biology techniques and cloned into either pBluescript KS+ for *in vitro* transcription or pcDNA3.1+ for cell transfection. ScaRNA9 mutant constructs were obtained by performing site-directed mutagenesis of either pcDNA3.1+ or pBluescript KS+ scaRNA9 WT using the Q5 Site-Directed Mutagenesis Kit (New England BioLabs, Ipswich, MA, USA) following the manufacturer's protocol. Primers, obtained from Integrated DNA Technologies (Coralville, IA, USA), and restrictions sites used in the cloning and mutagenesis procedures are detailed below. Note that endogenous scaRNA fragments are only faintly visible (Poole et al., 2016), thus the majority of the detected full-length and fragment scaRNA is from the processing of ectopically expressed primary-scaRNA. DNA transfections were conducted using FuGene HD (Promega, Madison, USA) according to the manufacturer's protocol.

### Cloning and mutagenesis primers

To clone WT scaRNA2, Forward 5′-GGCGGATCCGTTTTAGGGAGGGAGAGCGGCCTG-3′ and Reverse 5′-TATGGAATTCGGAAAGTGGGAGGAAAGTATATC-3′ primers were used. The underlined sequences denote a *Bam*HI and an *Eco*RI site, respectively. To clone WT scaRNA9, Forward 5′-GCCGAATTCTTAAGTTATGCTGTGGAGGAAG-3′ and Reverse 5′-GGCGCGGCCGCTTTCATAACTTAAAAGGCTCC-3′ primers were used. The underlined sequences denote an *Eco*RI and a *Not*I site, respectively. To clone scaRNA9 3′ ext, Forward 5′-GGCGGATCCCTTTCTGAGATCTGCTTTTAGTGA-3′ and Reverse 5′-GGCGAATTCAACAGTTGCTGAAGATAATGG-3′ primers were used. The underlined sequences denote a *Bam*HI and an *Eco*RI site, respectively. To clone WT scaRNA17, Forward 5′-TATGGGATCCAGAGGCTTGGGCCGCCGAGCT-3′ and Reverse 5′-TATGGAATTCGTGTTTGAAAAGCAGGATTCTAG-3′ primers were used. The underlined sequences denote a *Bam*HI and an *Eco*RI site, respectively. The following primers were used for scaRNA9 mutagenesis: Mutant #2, Forward 5′-ATGGGTTTCTACACTTGACCTG-3′ and Reverse 5′-GCTCAATAGTTACAAAGATCAGTAGTAAAAC-3′; Mutant #3, Forward 5′-TGCTGGGGTTGGTGATTT-3′ and Reverse 5′-AAGCTTTCACTTCTGAGCTCAGGTC-3′; Mutant #4, Forward 5′-GGATCCGTGGTATGATATTCCCCTTAATTTC-3′ and Reverse 5′-AAAACCCCAAATCACCAACC-3′; Mutant #5, Forward 5′-ATATTCCCCTTAATTTCTAGGC-3′ and Reverse 5′-AAGCTTTCACTTCTGAGCTCAGGTC-3′.

### *In vitro* Drosha/DGCR8 processing assay

*In vitro* processing assays were conducted using Drosha/DGCR8 immunoprecipitates. Myc-tagged Drosha and FLAG-tagged DGCR8 plasmids were obtained from Addgene (Watertown, MA, USA). HeLa cells were co-transfected with myc-Drosha and FLAG-DGCR8 DNA for 24 h. After 24 h, cells were washed with PBS and harvested using a KCl lysis buffer (20 mM Tris-HCl, 180 mM KCl, 0.2 mM EDTA) followed by sonication with a Fisher Scientific sonic dismembrator (model 100) six times for 5 s each using the output setting of 1, and finally centrifuged at 12,000 rpm for 15 min at 4°C. The FLAG-DGCR8/Drosha complex was immunoprecipitated using anti-FLAG-M2 affinity agarose beads (Sigma Aldrich, St. Louis, MO, USA) overnight and washed three to five times with KCl lysis buffer. The precipitated complex was then incubated for 90 min at 37°C with RNA substrate, along with 6.4 mM MgCl_2_, RNase inhibitor (ThermoFisher Scientific, Waltham, MA, USA), and RNase-free water in a total volume of 30 µl.

Control reactions include substrate incubated with water alone and substrate incubated with FLAG beads from non-transfected cell lysate. The RNA was then isolated using Tri-Reagent (Molecular Research Center, Cincinnati, OH, USA) following the manufacturer's protocol and subjected to Northern blotting using 5′ DIG probes specific for full-length scaRNA and the processed fragment, previously described (Poole et al., 2017), as follows: scaRNA9 mgU2-30 (5′- TAGAAACCATCATAGTTACAAAGATCAGTAGTAAAACCTTTTCATCATTGCCC-3′) and mgU2-19 (5′-GTAGACTGGAAAGACTTCTGATGCTCAGATTTGGCTAGTTTCATCATTGA-3′); scaRNA2 mgU2-61 (5′-AGTGGCCGGGGACAAGCCCGGCCTCGTCTATCTGATCAATTCATCACTTCT-3′); scaRNA17 mgU4-8 (5′-AACTCAGATTGCGCAGTGGTCTCGTCATCA-3′). DIG labeled probes were obtained from Integrated DNA Technologies (Coralville, IA, USA).

RNA substrate used in the *in vitro* processing assay was generated by the following method. First, scaRNA constructs in pBS KS+ were linearized at the 3′ end with *Eco*RI (for scaRNA2 and scaRNA17) or *Hind*III (for scaRNA9) and then gel purified using the QIAquick Gel Extraction Kit (Qiagen, Hilden, Germany). Linearized DNA was subjected to *in vitro* transcription with the Megascript T7 Transcription Kit (Thermo Fisher, Waltham, MA, USA). Lastly, the *in vitro* generated RNA was gel purified using the ZR-small RNA PAGE Recovery Kit (ZYMO Research, Carlsbad, CA, USA).

### Northern blotting

Total RNA was isolated using TRI-Reagent (Molecular Research Center, Cincinnati, OH, USA). For total RNA, typically 10-15 μg was run on a 6% denaturing polyacrylamide gel (Invitrogen, Carlsbad, CA, USA) in 1X Tris-Borate-EDTA (TBE) at 200 V. The gel was then washed in 1X TBE and then transferred onto a positively charged nylon membrane (Invitrogen, Carlsbad, CA, USA) with the iBlot Gel Transfer device (Life Technologies, Grant Island, NY, USA) using program 5 for 5 min. After transfer, the membrane was rinsed in ultrapure water, allowed to dry, and then subjected to a UV cross-linker (UVP, Upland, CA), at a setting of 120,000 μJ/cm2. The membrane was then placed in a hybridization bottle and pre-hybridized using Ultrahyb Ultrasensitive Hybridization buffer (Ambion Life Technologies, Grand Island, NY, USA) for 30 min at 42°C in a hybridization oven. The DNA oligo probes used for the detection of full-length and fragment scaRNAs 2, 9 and 17 were 5′ DIG labeled and previously described (Poole et al., 2017). Membranes were then prepared for detection using the DIG Wash and Block kit (Invitrogen, Carlsbad, CA, USA) following the manufacturer's suggested protocol with the Anti-DIG antibody used at 1:10,000. Detection was carried out using CSPD (Roche, Mannheim, Germany) following the manufacturer's suggested protocol. Blots were imaged using a Chemidoc imager (Bio-Rad, Hercules, CA, USA). Where noted, adjustments to images were made using the transformation settings on QuantityOne software and applied across the entire image.
